# Optimization of Power Consumption Associated with Surface Roughness in Ultrasonic Assisted Turning of Nimonic-90 Using Hybrid Particle Swarm-Simplex Method

**DOI:** 10.3390/ma12203418

**Published:** 2019-10-18

**Authors:** Navneet Khanna, Jay Airao, Munish Kumar Gupta, Qinghua Song, Zhanqiang Liu, Mozammel Mia, Radoslaw Maruda, Grzegorz Krolczyk

**Affiliations:** 1 Institute of Infrastructure, Technology, Research, and Management, Ahmedabad 380026, India; navneetkhanna@iitram.ac.in (N.K.); jay.airao.16mm@iitram.ac.in (J.A.); 2 Key Laboratory of High Efficiency and Clean Mechanical Manufacture, Ministry of Education, School of Mechanical Engineering, Shandong University, Jinan 250061, China; munishguptanit@gmail.com (M.K.G.); ssinghua@sdu.edu.cn (Q.S.); melius@sdu.edu.cn (Z.L.); 3 National Demonstration Center for Experimental Mechanical Engineering Education, Shandong University, Jinan 250061, China; 4 Department of Mechanical Engineering, Imperial College of London, South Kensington, London SW7 2AZ, UK; mozammelmiaipe@gmail.com; 5 Faculty of Mechanical Engineering, University of Zielona Gora, 4 Szafrana St, 65-516 Zielona Gora, Poland; r.maruda@ibem.uz.zgora.pl; 6 Faculty of Mechanical Engineering, Opole University of Technology, 76 Proszkowska St, 45-758 Opole, Poland

**Keywords:** ultrasonically assisted turning, Nimonic-90, surface roughness, power consumption, optimization, nature inspired hybrid algorithm

## Abstract

These days, power consumption and energy related issues are very hot topics of research especially for machine tooling process industries because of the strict environmental regulations and policies. Hence, the present paper discusses the application of such an advanced machining process i.e., ultrasonic assisted turning (UAT) process with the collaboration of nature inspired algorithms to determine the ideal solution. The cutting speed, feed rate, depth of cut and frequency of cutting tool were considered as input variables and the machining performance of Nimonic-90 alloy in terms of surface roughness and power consumption has been investigated. Then, the experimentation was conducted as per the Taguchi L9 orthogonal array and the mono as well as bi-objective optimizations were performed with standard particle swarm and hybrid particle swarm with simplex methods (PSO-SM). Further, the statistical analysis was performed with well-known analysis of variance (ANOVA) test. After that, the regression equation along with selected boundary conditions was used for creation of fitness function in the subjected algorithms. The results showed that the UAT process was more preferable for the Nimconic-90 alloy as compared with conventional turning process. In addition, the hybrid PSO-SM gave the best results for obtaining the minimized values of selected responses.

## 1. Introduction

In this growing industrial world, the trend of modern materials, especially nickel based alloys, are prevalent in various sectors such as automobile, aerospace, marine etc. [1]. They are altogether expected in these manufacturing sectors because of their eminent characteristics such as high resistance to corrosion and excellent mechanical properties etc. [2]. These characteristics, however, result in enormous challenges in terms of high tool wear, low finishing, excessive forces etc. in machining of advanced materials [2,3]. Furthermore, the strict environmental policies and concerns are other challenges which must be addressed during the machining of advanced materials. For instance, Japan has established the basic “Energy Policy” which primarily focuses on energy related issues in manufacturing sectors. Similarly, the USA have introduced the special program on “Superior Energy Performance (SEP)” that provides a track in the field of sustainability development for manufacturing sectors. Likewise, the European countries have developed the ISO standards i.e., 5001 for regularization of energy standards in manufacturing sectors [4].

In order to follow the environmental concerns and ISO 5001 standards, the new technologies i.e., hybrid machining processes are considered as main drivers to support the working aspects of sustainability i.e., social, economic and environmental [5,6]. In a hybrid machining process, the material removal mechanism is totally different as compared with conventional machining processes. For instance, in the hybrid machining process, the material is removed with the main machining process while a secondary technique “assists” the material removal by improving the conditions of machining. In recent years, the ultrasonic assisted turning (UAT) process has been termed as one such hybrid process that uses ultrasonic vibrations for the cutting action [5]. In this hybrid process, the interaction of the cutting tool and the workpiece directly takes place and the material is removed under the action of micro chipping [6]. Furthermore, the vibrations of the tool produce some surface texturing effect on the workpiece [7] and thereby good surface finishing, dimensional accuracy and low tool wear are obtained during machining [8]. The efficiency of UAT has been noted by various former researchers. Some of their works are presented here. In the first study of Maurotto et al., it has been seen that the cutting forces produced in the UAT process are significantly less as compared with the conventional dry turning (CT) of Ti-15333 and Ni-625 alloys [9]. In another similar work, the cutting forces were analyzed by Ahmed et al. during machining of Inconel-718 [10]. It was found that the cutting forces induced during CT were 130–140 N whereas; in UAT process were 60–95 N. In the same work, Maurotto et al. showed that the tangential as well as radial cutting forces were reduced up to 70%–80% while machining of Ti-15333 and Ni-625 which was claimed to be possibly due to ultrasonic softening in the base alloys in UAT [11]. It was also determined by the same authors that the cutting speed is the major factor in UAT process that effects the cutting forces when compared to CT [12]. Silberschmidt et al. analyzed the surface roughness values in machining of Inconel-718 and Ti-15333. The comparison was also made between UAT and CT process [13]. Similarly, Zhong and Lin found that the surface roughness improves by 15% with high amplitude as compared to lower amplitude with UAT because of the ironing effect in aluminum metal matrix composites [14]. Moreover, Nath and Rahman [15] studied the effect of frequencies, amplitude and cutting speed on cutting forces values. They concluded that the cutting force generated during the UAT is dependent on tool–workpiece contact ratio (TWCR). In the same context, Vivekananda et al. [16] implemented the Taguchi design of experiment process to optimize the cutting force and surface roughness values in the UAT process.

From the comprehensive state of art review, it has been interestingly noticed that UAT is a very tremendous technology in the modern arena of manufacturing sectors. However, its application is only limited to the cutting forces and tool life while machining of other nickel-based alloys i.e., Inconel 718, whilst surface roughness and power consumption are overlooked. Moreover, the study of UAT of Nimonic-90 has never been performed to the best of the authors’ knowledge. Although previous investigations have shown that UAT improves the machinability of Inconel 718 alloy, these results cannot be directly extended to Nimonic-90 alloy. Thus, to bridge this gap, a series of UAT experiments were conducted and the surface roughness and power consumption were investigated and analyzed for Nimonic-90 alloy. In addition, the literature reveals that the performance of UAT is highly dependent upon its process parameters because a large number of process parameters are involved in the ultrasonic assisted turning process. Therefore, the best parameters settings are required to enhance the machining performance of the UAT process. Various types of optimization methods i.e., conventional and advanced methods, are currently available in the literature that improve the process efficiency by changing the input and output settings [17,18,19]. In the conventional methods Taguchi, signal to noise (S/N) ratio, analysis of variance, regression, desirability analysis etc. have been introduced to solve the optimization issues [20,21,22]. However, the conventional methods are subjected to some issues, such as the lack of targeting the global optimal solution with these methods, which may result in low accuracy and non-robustness of the results. With these limitations, they are still used in the machining of different materials. For instance, Shokrani et al. [23] used the Taguchi method to optimize the process parameter in cryogenic milling of Ti64 titanium alloy. Islam et al. [24] compared the traditional and Taguchi method in terms of efficiency to analyze the surface roughness values. Ezilarasan et al. [25] used the Taguchi method to discuss the effect of input variables on surface roughness values while machining Nimonic C-263 alloy. Makadia and Ashvin [26] minimized the surface roughness values in machining AISI 410 steel by using the response surface methodology (RSM) method. Bhushan [27] optimized the parameters for minimum power consumption and improve the tool life during machining of 7075 Al alloy SiC particle composites with the help of response surface methodology.

Apart from these optimization methods, the various types of advanced methods such as evolutionary algorithms, nature inspired hybrid algorithms and intelligent methods are well implemented in literature to solve optimization problems [17,18,19]. The major benefits of these advanced methods are that they accurately achieve the global optimal solution with a small interval of time [17,28]. They are generally presented in the MATLAB code and the objective or fitness function is required to run the program. Moreover, the single or multi-objective problems can be easily tackled with these advanced optimization methods. Numerous studies have been available in the literature that clearly represent the application of advanced optimization algorithms in the machining sector. In the first study, Singh et al. used the two algorithms i.e., particle swarm and bacterial foraging for optimization of cutting parameters in minimum quantity lubrication (MQL) assisted milling of commercially available Inconel-718 alloy [19]. Likewise, Sahu and Andhare used the three advanced algorithms i.e., teaching learning based optimization (TLBO), Jaya algorithm and genetic algorithm (GA) and one conventional method (RSM) to solve the optimization problem of Ti–6Al–4V alloy [29]. They suggested that the performance of the TLBO and Jaya algorithms is better than GA. In another optimization study, Sathish applied the hybrid bee colony cuckoo search (BCCS) and RSM approach in non-conventional machining of Nimonic-263 alloy [30]. In similar work, Gupta et al. implemented the particle swarm optimization (PSO) and bacterial foraging optimization (BFO) while turning titanium (grade-2) alloy under nano-fluid cutting conditions [17]. The performance was also compared with the traditional optimization method i.e., RSM. It has been noted that the PSO and BFO work more efficiently than the RSM methods and significantly enhance the process performance. Furthermore, Rao and Venkaiah used the PSO and RSM to optimize the machining parameters of Nimonic-263 alloy [2].

Thus, as per the availability of current research survey, it has been clearly noted that the machining performance of any material is highly improved with the implementation of advanced algorithms. For instance, Sahu and Andhare, Sathish, Singh et al. and Gupta et al also presented similar findings in machining of Inconel, titanium and Nimonic-based alloys [15,16,26,27]. Still, with all these hard efforts, no research work is available in the literature which shows the application of advanced algorithms in ultrasonic assisted turning of Nimonic-90 alloy for power optimization. Therefore, this research work firstly reported the application of nature inspired hybrid algorithm i.e., particle swarm optimization (PSO) method hybridized with the simplex method (SM) during ultrasonic assisted turning of Nimonic-90 alloy. The input parameters considered were cutting speed, feed rate, depth-of-cut and frequency of the cutting tool used. A Taguchi L9 orthogonal array with three repetitions were used as an experimental design and the power consumption and surface roughness values were optimized with these implemented algorithms. The complete detail of this experimental work complemented with the optimization details are presented in the subsequent sections.

## 2. Materials and Methods

### 2.1. Ultrasonic Assisted Turning (UAT) Process

**Figure 1:**
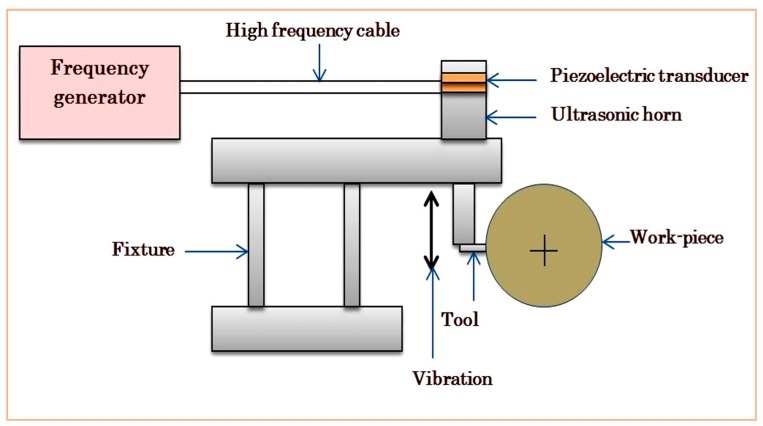
Schematic of ultrasonically assisted turning set-up.

The simplified view of the UAT process is exhibited in Figure 1. The main components of the UAT machine were the frequency generator, piezoelectric transducer and the horn. The frequency generator created the electrical signal which was then converted into a mechanical signal by a piezoelectric transducer. Then, these mechanical signals propagated through the ultrasonic horn to the cutting tool.

The major aim of this ultrasonic horn was to amplify the vibrations to reasonable magnitudes. Well-known analytical relations exist which are used to facilitate the horn design. For example, the length of stepped horn *(L)* is determined using $L=\frac{c}{2f}$. Here, *f* is frequency and *c* is the speed of sound in the medium (horn material) which depends on the modulus of elasticity *(E)* and density *(ρ)* of the material as shown in$\;c=\sqrt{\frac{E}{\rho }}$. Titanium, aluminum, mild-steel etc. are popular choices for horn material. In our study, mild steel was used to manufacture the horn [31].

### 2.2. Workpiece and Tool Material

The workpiece materials used were Nimonic-90 alloy. They were precipitation strengthened nickel base super alloys of extra high mechanical properties with corrosion resistance. Nimonic-90 is typically used in extreme stress applications such as turbine blades, hot working tools, exhaust re-heater, disc and high-temperature springs. The chemical composition of Nimonic-90 alloy is shown in Table 1.

Similarly, for performing the turning experiments, chemical vapour deposition (CVD) coated carbide inserts with a layer of TiC, Al_2_O_3_ and TiN were used. The technical specification of the tool is presented in Table 2. Note that the length of cut used was 50 mm and for each cut a fresh cutting edge was used.

### 2.3. Process Parameters

The selection of input parameters was based on the experience of local small and medium-sized enterprises (SMEs), specially involved in machining of Nimonic-90. The selected reposes i.e., cutting speed (V), feed rate (F), depth of cut (DOC) and frequencies (f) chosen for the experimental study are shown in Table 3. Note that for the UAT process, $V_{c}=2\pi af>V=\pi DN\;$should be satisfied where a is amplitude, f is frequency, D is diameter of the workpiece, and N is rotating speed (rpm) of the spindle. If the cutting velocity V, exceeds the critical cutting velocity, $V_{c}$, the UAT process effectively reduces to a conventional machining process.

### 2.4. Design of Experiment

**Figure 2:**
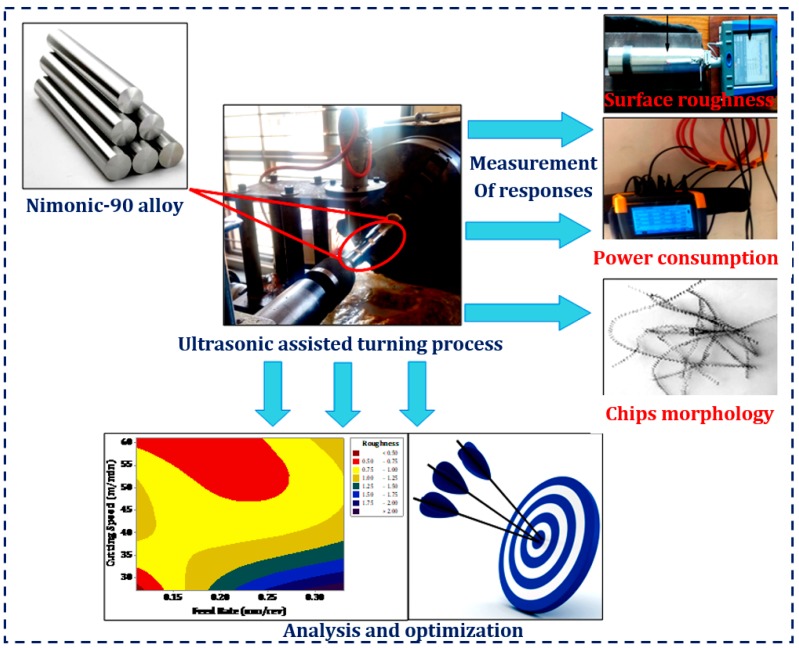
Experimental procedure with complete details.

The turning tests were carried out by considering a Taguchi L9 orthogonal array (as presented in Table 4). According to this design, a total of nine experiments with three repetitions were conducted. Then after, the analysis of variance (ANOVA) test (using the Minitab 18 software, State College, PA, USA) was implemented on the experimental results. The experimental procedure with complete details is exhibited in Figure 2.

### 2.5. Measurement of Responses

In this study, two important machining indices i.e., the average surface roughness ($R_{a}$) and power consumption (P) were measured after each experiment. For measurement of surface roughness values, the Taylor Hobson Surface roughness tester (AMETEK, Leicester, UK) was used. The power consumption (P) after each cut was measured with fluke power analyzer 435 series.

## 3. Nature-Inspired Algorithms

This section describes the overview of implemented algorithms i.e., particle swarm optimization, simplex method and hybrid PSO-SM, respectively. The working principle and procedure are discussed as per the following.

### 3.1. Particle Swarm Optimization (PSO)

PSO is categorized as the nature inspired-optimization algorithm in which the problem of linear and non-linear programming has been successfully solved [32]. Two paramount terms i.e., particles position as well as velocity has been recognized in the status of PSO method [18]. The *ith* particle position and its velocity in the d-dimensional search space are well described with the following Equations (1) and (2), respectively.

(1) $$X_{i}=\left[x_{i,1,}x_{i,2,}\ldots \ldots \ldots x_{i,d}\right],$$

(2) $$V_{i}=\left[v_{i,1,}v_{i,2,}\ldots \ldots \ldots v_{i,d}\right],$$

where, *X_i_* and *x_i_* up to the *d*th terms are integral values related to the position of particles, *V_i_*, *v_i,_*_1_, … *v_i,d_* are the velocity values of particles.

In the PSO method, every particle consists of an ideal position (*pbest*) also known as location with respect to the individual ideal values at particle interval of time, *t*. The *pbest* ($Pb_{i}$) is calculated with the help of Equation (3).

(3) $$Pb_{i}=\left[pb_{i,1,}pb_{i,2,}\ldots \ldots \ldots pb_{i,d}\right].$$

Similarly, the global ideal value (*gbest*) of each particle is termed by Pbg that generally shows the best or ideal particle at time, *t*. After that, the Equation (4) is used to evaluate the updated velocity of every particle [33,34].

(4) $$v_{i,j}\left(t+1\right)=wv_{i,j}\left(t\right)+\;c_{1}r_{1}\left(pb_{i,j}-x_{i,j}\left(t\right)\right)+c_{2}r_{2}\left(pb_{g,i}-x_{i,j}\left(t\right)\right),\;j=1,2,\;\ldots \;d,$$

where, *v_i,j_*(*t +* 1), *x_i,j_*(*t*) are function values, $c_{1}$ and $c_{2}$ represent coefficient values, inertia factor is denoted by w, $r_{1}$ and $r_{2}$ are termed as random variables having values of (0, 1). Therefore, Equation (5) is used to update the position of every particle.

(5) $$x_{i,j}\left(t+1\right)=x_{i,j}\left(t\right)+v_{i,j}\left(t+1\right),\;j=1,2,\ldots \;d.$$

In general, the $v_{i,j}$ in the Equation (4) of every component is expressed in terms of $-v_{max}\;to\;v_{max}$. These values are used to control the tremendous routing of external particles during the search space. Then, the particles follow Equation (5) and the positions of particles are updated towards a newer position [35]. Hence, the process is worked again and again until a global optimal solution is achieved.

### 3.2. Simplex Method (SM)

In this paper, the simplex method modified by Nelder and Mead, in 1965, was used to tackle the constrained and unconstrained optimization problems [35]. In this method, firstly the n input values at the polyhedron phase is considered and further the n+1 points with $R^{n}$ series are applied to establish the mathematical model. After that, the initial simplex changes its position i.e., moves, contracts and expands because of their series of primary geometric transformations, respectively. Then, the lower which also knows as the worst point $\left(X_{w}\right)$ at every iteration is calculated by ordering and classifying the vertices values as $X_{1},X_{2},\ldots ,X_{n},X_{n+1},$ so that the solution is$\;f\left(X_{1}\right)<f\left(X_{2}\right)<\ldots f\left(X_{n}\right)<f\left(X_{n+1}\right)$.

The value of objective function in the simplex method is decided as per the user requirements i.e., whether to minimize it or maximize it. For minimization, the variable with the largest objective value is used for a new reflection and the ideal point value has been placed approximately in the negative gradient direction [36]. For instance, $X_{1}$ represents the ideal point, $X_{n+1}$ is termed as the worst or lowest point, $X_{n}$ describes another worst point and so on. Moreover, the centroid point $\left(X_{c}\right)$ of the n ideal solutions excluding $X_{n+1}$ is calculated. In the end, the lowest or worst point is reflected in Equation (6) and latest point $\left(X_{r}\right)$ is obtained. In addition, at this point, if the function i.e., $f\left(X_{1}\right)\leq f\left(X_{r}\right)<f\left(X_{n}\right)$ and boundary conditions are not desecrated, then the reflection takes place at an ideal region of search space and the replacement of the lower or worst point $X_{n+1}$ is made with$\;\left(X_{r}\right)$, hence the iteration stops working. Similarly, the other behavior i.e., expansion, contraction, shrinkage, movement of variables are calculated by Equations (7)–(10). Note that, the objective or fitness function is computed at each point of the method and the complete process is processed again and again until the final solution has been achieved.

(6) $$X_{r}=X_{c}+\rho \left(X_{c}-X_{n+1}\right),$$

(7) $$X_{e}=X_{c}+\gamma \left(X_{r}-X_{c}\right),$$

(8) $$X_{cont1}=X_{c}-\gamma \left(X_{c}-X_{n+1}\right),$$

(9) $$X_{cont2}=X_{c}+\gamma \left(X_{r}-X_{c}\right),$$

(10) $$P_{i}=X_{1}+\sigma \left(X_{i}-X_{1}\right),\;i=2,\ldots ,\;n+1,$$

where, $\left(X_{i},P_{i},\ldots ,P_{n+1}\right)$ reflects the new vertices, $X_{e}$, $X_{r}$, $X_{cont1}$ and $X_{cont2}$ shows the behavior at expansion, contraction and stretching.

### 3.3. Hybrid PSO-SM

PSO is known as the nature inspired algorithm, whereas the simplex method is referred to as an intelligent strategy that is effectively used to solve linear and non-linear problems [36,37]. The main aim of hybridization is to merge the advantages of both methods [38,39]. In addition, the searching of PSO is performed as per the Equations (1) and (2) and integrating PSO with simplex method may enhance the capacity to search the space towards the global optimal solution [36,37]. For instance, Equations (6)–(10) are used to show the behavior i.e., $X_{e}$, $X_{r}$, $X_{cont1}$ and $X_{cont2}\;$and they are further divided by the swarm characteristics with their vector values i.e., $X_{i}\left(N_{i},x_{i},C_{1i},C_{2i}\right),\;upto\;i\;=\;1,\ldots ,\;n\;+\;1$, where n is referred to the PSO parameters and $N_{i}$ is an integer value.

The hybridization is performed in two ways: (1) the staged pipelining type in which each population size of PSO is processed by the stochastic optimization method and the simplex search is used for the improvement. Similarly, (2) the additional-operator type hybrid method in which the simplex search is directly applied to the population values and the probability of improvement is targeted by the user [36,37]. Therefore, in the paper, the hybridization of both methods is made by the staged pipelining method. The complete process is described below:

1. Initialization Step: The ideal positions of initials particles, generations of random *N* particles are selected and evaluated.
2. Repairing Step: The particles have been repaired that affects the boundary conditions by expressing the worst solution towards the ideal solutions. Moreover, terminate the damaged particles.
3. Searching Step: Equation (2) is used to search the individual position of each particle. The step is to select the better or ideal position and evaluate them.
4. Ranking: The obtained solution has been ranked according to their best fitness values, from the Equations (1) and (2).
5. Selection Step: Equation (2) is used to select the better position of each particle and the generation of ideal solution has been obtained.
6. Generation Step: Further, the D+1 points have been selected from the population based ranking solution and the initial simplex is well generated.
7. Simplex Method: It is applied on the highest N+1 particles and (N+1)th has been updated.
8. Step 6 is replaced with Step 7 i.e., simplex method, so the best solution has been memorized, until the final solution has been achieved.

## 4. Results and Discussion

This section represents the prominent part of the paper. The statistical analysis was performed with the ANOVA test followed by influence of process parameters and estimation of optimum quality characteristics. The details of these analyses are discussed below:

### 4.1. Statistical Testing

In this analysis, the relationship between input variables and responses were made from the experimental results. The individual results of selected responses are shown in Table 4. The present statistical analysis was performed at 95% confidence interval (CI), which means at α = 0.05 significance level. Further, the F-tests and *p*-value tests (less than 0.05) at 95% CI were performed on experimental values and are displayed in Table 5 and Table 6, respectively. These tests were used to represent the effect of process parameters on responses. For instance, if the F-tests had high values, the more an effect was shown on the process variable. Moreover, the total effect was calculated by the percentage contribution values in respective tables.

Further, Table 5 shows the experimental results during ultra-sonic assisted turning of Nimonic-90 alloy under different cutting conditions. From the surface roughness analysis, i.e., the F-test showed that the maximum value i.e., 347.83 was for feed rate which meant the feed rate had the highest effect or highest contribution of 44.065% on surface roughness values followed by depth of cut (24.163%), cutting speed (17.051%) and frequency values (13.586%). A similar trend is observed by Reference [15] in machining of titanium alloy. Similarly, from power consumption analysis, the cutting speed (39.798%) had highest effect on power consumption followed by frequency (19.32%), depth of cut (16.648%) and feed rate (7.05%), respectively. In addition, the *p*-value test showed that the developed models were statistically significant for selected responses.

### 4.2. Influence of Process Parameters

Surface roughness: The contour effect plots were drawn to demonstrate the influence of different machining conditions on surface roughness values. From the previous statistical analysis, it was clearly noticed that the feed rate highly affected the surface roughness values. This statement is purely justified with the following Equation (11) which shows that the surface roughness is directly proportional to the square of feed rate as per the basic relation.

(11) $$R_{a}=\frac{f^{2}}{8r}$$

where, $R_{a}$ is arithmetic roughness, *f* defines as a feed rate in mm/rev, *r* represents the nose radius in mm.

**Figure 3:**
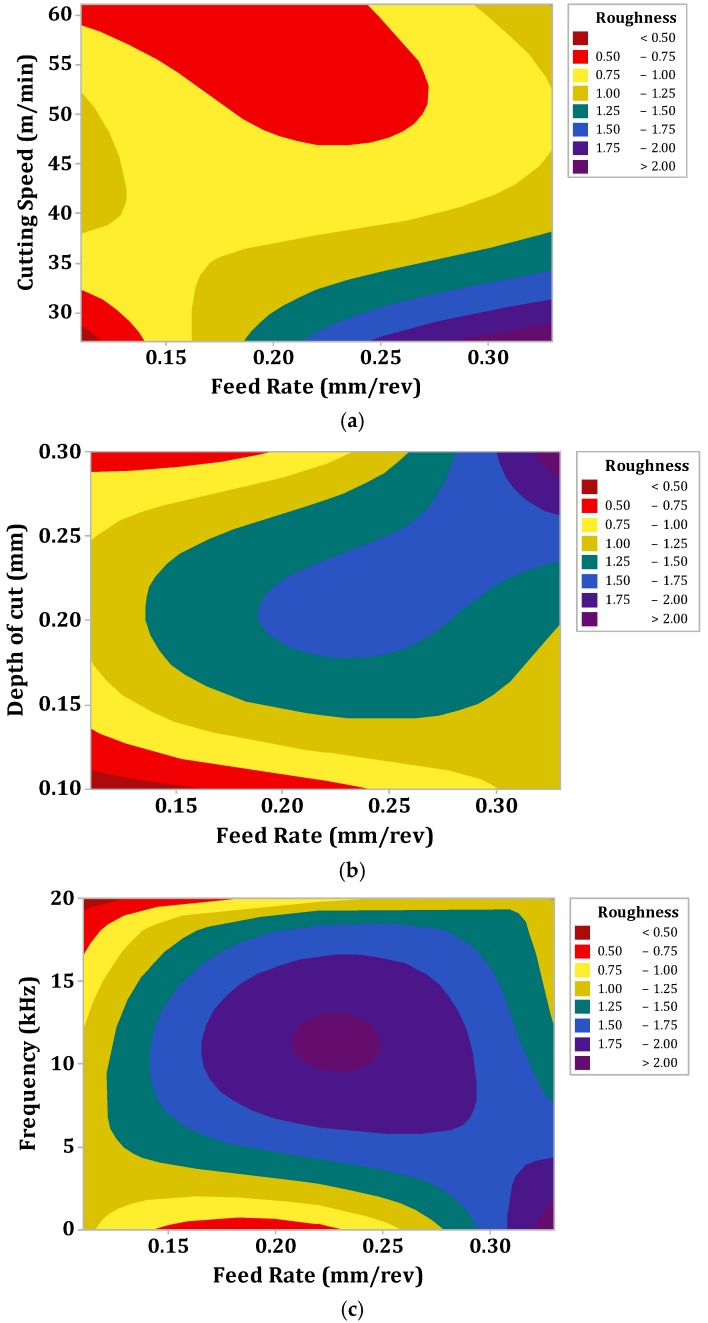
Influence of machining parameters on surface roughness values (**a**) Cutting speed vs. feed rate, (**b**) depth of cut vs. feed rate, and (**c**) frequency vs. feed rate.

Therefore, the contour effect plots showing maximum effect of feed rate on surface roughness values were used in this work (as depicted in Figure 3a–c). Figure 3a claims that the surface roughness was minimum at lower values of cutting speed and feed rate. However, it swelled with the rise in feed rate, whereas it dwindled with the change in cutting speed values. The trends of these results were verified with the mechanism given in the Equation (11). Further, the increase in cutting speed lowered the formation of built-up edges at the tool surface. As a consequence, the low surface roughness values were achieved at higher values of cutting speed. Practically, these results may not fulfill the favorable conditions because high surface roughness values are not recommended to achieve the sound machining characteristics. The similar findings were reported by Reference [6]. Similarly, Figure 3b demonstrates the contour effect plot of depth of cut vs. feed rate. The observation results of these plots claim that the lower values of surface roughness were achieved at low depth of cut values and once the depth of cut was changed i.e., from minimum to maximum, undesirable machining surface characteristics were achieved. This is a very interesting fact as the tool area had a higher amount of contact with the subjected workpiece at higher depth of cut values and thereby more frictional heat was produced at the cutting zone. Besides, the heat was not dissipated in the proper manner from the cutting zone because of the intrinsic characteristics i.e., poor thermal conductivity of Nimonic-90 alloy. This high temperature resulted in high affinity to tool materials which may cause the welding of micro-particles of the workpiece to the cutting tool and consequently, reduces the surface finishing values [5].

**Figure 4:**
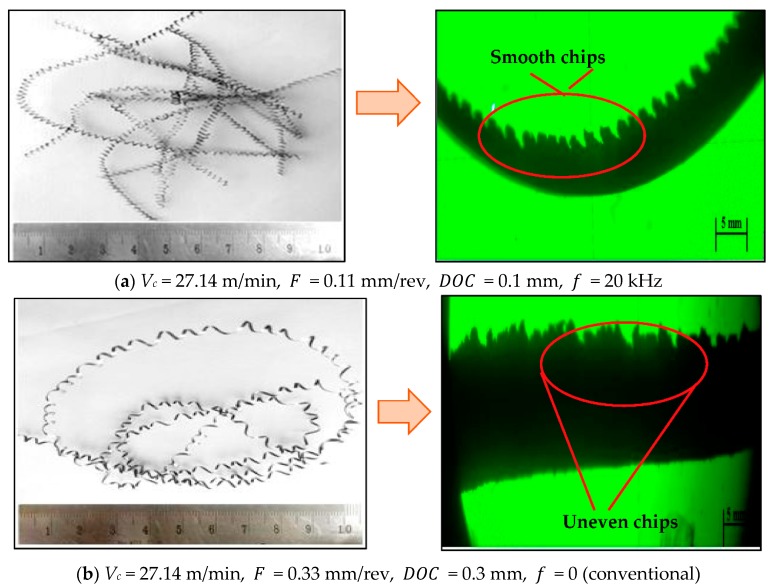
Macrographs and chip formed during machining of Nimonic-90 alloy under different conditions, (**a**) smooth and short chips, (**b**) longer chips.

**Figure 5:**
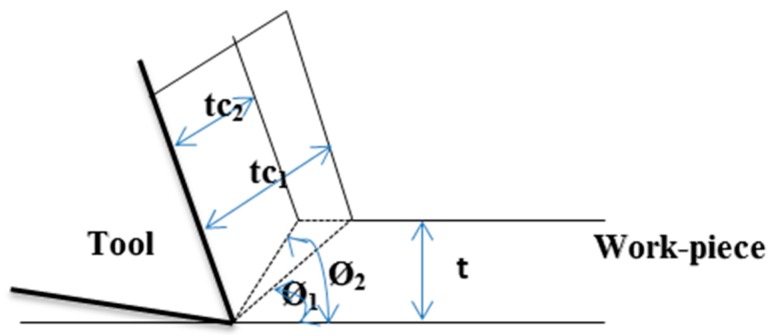
Effect of small (${Ø}_{1}$) and large (${Ø}_{2}$ ) shear angle on chip thickness ($t_{c}$ ) and length of shear plane for a given tool and un-deformed chip thickness (t ) [26].

Lastly, Figure 3c depicts the contour effect plot of frequency vs. feed rate. This plot exhibits that the lower value of surface roughness was achieved at the higher frequency of cutting tool i.e., at 20 kHz frequency. Moreover, this plot shows that the conventional machining process produced a higher value of surface roughness and it decreased with the change in frequency of cutting tool i.e., 20 < 18 < 0, respectively. This is generally related with the fact that the chips produced in the UAT process are smooth, thinner and shorter when compared to those obtained from conventional turning process (as shown in Figure 4). These smooth and short chips do not stick to the workpiece material and hence reduce the surface roughness values. Moreover, in the conventional turning process, longer chips are produced and these longer chips are undesirable which lead to entanglement of chips with the cutting tool and produces the rough surface. Further, the concept of smooth, thinner and shorter chips are directly related with shear angle and in the case of UAT it is increased. Hence, this increase in shear angle resulted in the decrease in chip thickness and as a consequence a good surface was produced with the increase in frequency of cutting tool (see Figure 5). In addition, the micrographs of chips during UAT and CT processes are presented in Figure 4. From this micro-graph analysis, it was interestingly seen that chips produced during the UAT process were regular while those produced from CT showed irregularities which manifested the poor surface quality of the machined surface. This is subjected to reason that when high frequency vibrations are exposed on cutting tool inserts, the removal of chips takes place because of the effect of vibrations and impact [40]. Moreover, the velocity of the stress wave, because of vibration of the cutting tool, produced a great impact on cutting velocity and hence, the inner stress broke the chips into small segments, and as a result soft, small and smooth chips were produced in the UAT process. Further, the tool work contact ratio was decreased with the increase in frequency of the tool. As a result, the temperature was reduced in the cutting zone because of the aerodynamic lubrication effect and hence the surface finishing was improved in the UAT process [5].

Power consumption: The power consumption is a very prominent aspect, especially during machining of hard-to-machine materials. It is also more important from a sustainable or environment point of view as it is directly related to the cutting forces, machine deformation and efficiency etc. Theoretically, it is a multiplication of main cutting force with the cutting speed values. Equation (12) is used to calculate the power consumption during each cut.

(12) $$p=\frac{F_{c}\times V_{c}}{60000},$$

where, p = power consumption in watts, $F_{c}=\;k_{c}\times a_{e}\times f$ is main cutting force in Newton and $V_{c}$ is the cutting speed in m/min, ae is the depth of cut in mm, f is the feed rate in mm/rev and $k_{c}$ represents as a specific cutting energy coefficient, respectively. Therefore, the power consumption is modified with the following Equation (13). This combination directly states that the power consumption has the direct effect on cutting speed, feed rate and depth of cut. Hence, all these subjected parameters were considered during the power consumption analysis [40].

(13) $$p=\frac{k_{c}\times a_{e}\times f\times V_{c}}{60000}.$$

**Figure 6:**
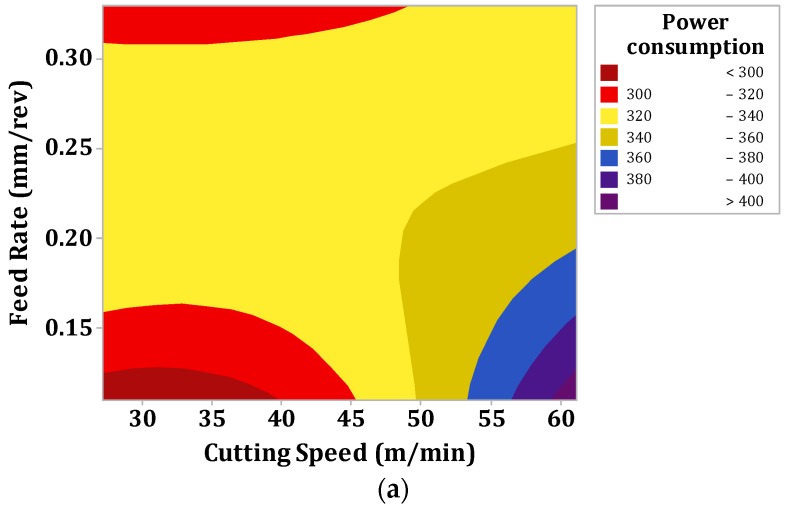

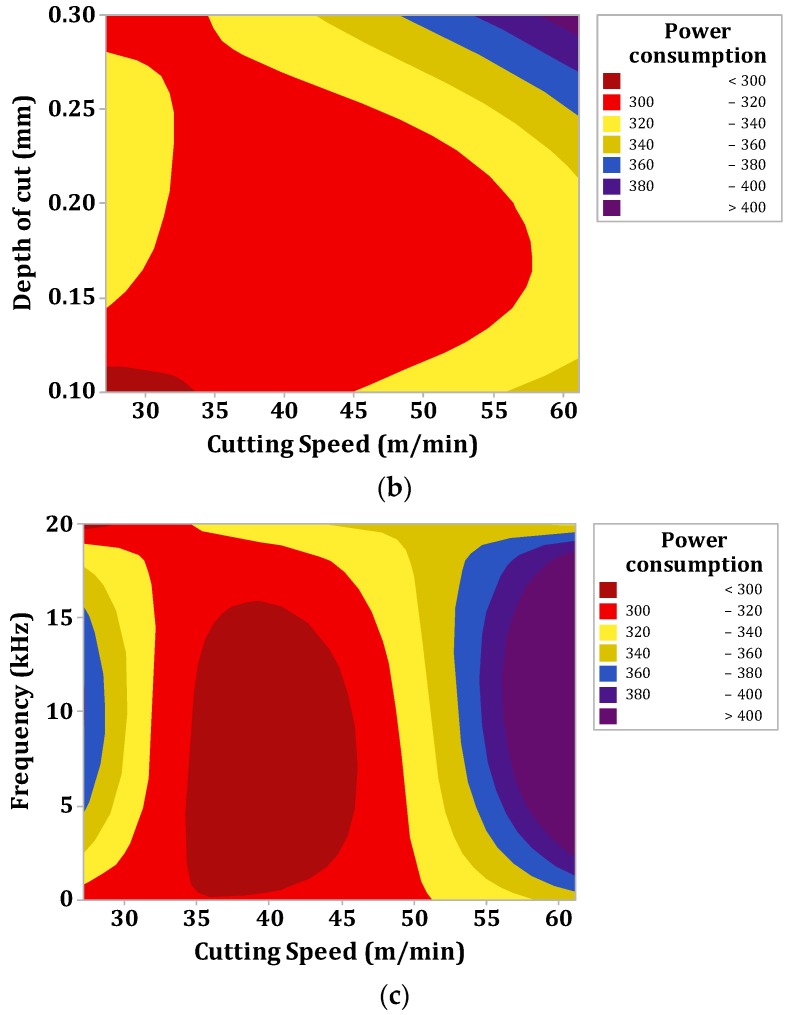
Influence of machining parameters on power consumption values (**a**) Feed rate vs. cutting speed, (**b**) depth of cut vs. cutting speed and (**c**) frequency vs. cutting speed.

Figure 6a–c depict the contour effect plots (a) cutting speed vs. feed rate, (b) cutting speed vs. depth of cut and (c) cutting speed vs. frequency. Figure 6a states that the combination of high cutting speed and low feed rate values are responsible for the high-power consumption. Equation (12) already justifies this statement.

This is a true fact that describes the enhancement of power consumption values with the increase in cutting speed because the values of power consumptions were totally dependent on the spindle’s rotation per minute (RPM) and the increased in spindle speed consumed more power from the motor. Similarly, the increase in the feed rate value demonstrated the lower power consumption values. This is the general machining fact that high feed rate values lead to low machining time and with this low machining time the tool engagement time is reduced with the workpiece. Hence, low power is consumed during higher feed rates as compared with lower feed rate values.

After that the effect of depth of cut along with the cutting speed is presented in Figure 6b. From this analysis, it has been interestingly noted that the values of power consumption were slightly increased with the depth of cut. In addition, the value of power consumption decreased with the increase in frequency of cutting tool, as depicted in Figure 6c. In the UAT process, as the frequency increased the tool vibration period decreased and tool vibration period for the higher frequency was lower than tool vibration period for lower frequency. Consequently, the tool workpiece contact ratio for higher frequency was lower than tool workpiece contact ratio for lower frequencies and with this a low tool workpiece contact ratio the tool engagement time was reduced with the workpiece. Hence, the slightly low power was consumed during higher frequency as compared with lower frequency values. Another possible reason for this behavior was the increase in shear angle during machining with higher frequency which led to a decrease in cutting forces and consequently power consumption.

### 4.3. Estimation of Optimum Quality Characteristics for Mono and Bi-Objective Optimization

The implemented algorithms were applied in two ways: (1) mono-objective (2) bi-objective. In mono-objective optimization, the process variables area was individually optimized in terms of input variables as well as responses. For this, the regression equations were directly used in the fitness function of algorithms. Whereas, in bi-objective optimization, one compromised or combined solution was derived for optimization of process parameters. The details of parameters initialization step followed by mono, bi-objective and algorithms confirmation are discussed below:

#### 4.3.1. Basic Parameters: Learning Parametric Setting for PSO and hybrid particle swarm-simplex (HPSO-SM)

The nature-inspired algorithms have some specific parameters i.e., maximum and minimum weight, constants ($W_{max},\;W_{min}$, *C*_1_, *C*_2_ and *H*) that explore its performance up to certain extent. In general, the role of these algorithm parameters is to decide the effectiveness of algorithm. The basic parameters used for PSO algorithm are shown in Table 7 and Table 8. These parameters are selected based upon the user’s experience and literature survey. For instance, in previously published work [41], the value of x is introduced in the range of 0–1.4, *C*_1_ and *C*_2_ are 2 and H is in the range of 5–10. Therefore, to effectively preserve the balance between local and global solution, the value of H is selected as 5. Besides, we have noticed that the selected parameters worked in a very efficient manner and significantly improved the efficiency of PSO. Moreover, the simplex method was also coupled with the PSO method and with this integration the performance characteristics with respect to the searching capability of these initial parameters were improved.

#### 4.3.2. Mono-Objective Optimization

In this section, the main aim was to determine the individual optimum parametric setting which showed the minimum values of responses. For this, initially the regression equations for individual parameter in terms of variables were developed and these equations were further used as a fitness function in the MATLAB code of algorithms. The boundary conditions (ranges of input parameters) and objective functions (in terms of regression equations) used in the MATLAB code are discussed below. Boundary conditions: cutting speed: 27.14 ≤ *V_c_≤* 61.14, feed rate: 0.11≤ *f ≤* 0.33, depth of cut: 0.1 ≤ *ae ≤* 0.3 and frequency: 0 ≤ *frequency* ≤ 20.

Objective functions:

(14) Ra = 0.595 − 0.01487 Vc + 3.85 f + 2.62 ae − 0.0186 frequency

(15) P = 231.0 + 1.671 Vc − 74 f + 178 ae + 0.74 frequency

**Figure 7:**
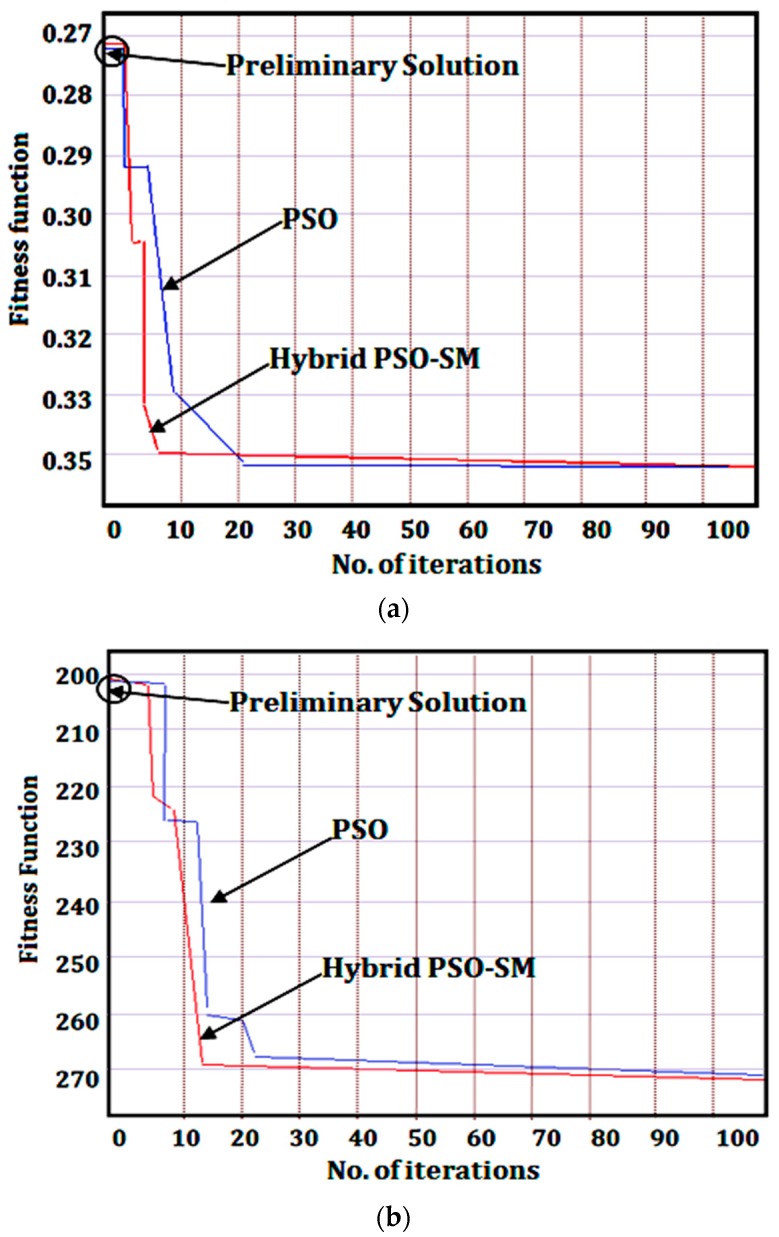
Convergence characteristics graphs for mono-objective optimization (**a**) minimum surface roughness value, (**b**) minimum power consumption value.

Based upon these boundary conditions and objective functions, the optimization by using the general PSO and hybrid PSO-SM method have been performed. The optimized values selected for surface roughness and power consumption are presented in Table 9. Similarly, the convergence characteristics graph of each factor is shown in Figure 7. From the generated results, the selected values were: $V_{61.14},\;F_{0.11},\;\;DOC_{0.1}\;and\;f_{20}$ for minimum surface roughness values (0.35 µm) and $V_{27.14\;},\;F_{0.33}$, $DOC_{0.1}$ and $f_{20}$ for minimum power consumption values (270 Watt), where the subscript represents the value of the respective cutting parameter, respectively.

#### 4.3.3. Bi-Objective Optimization

**Figure 8:**
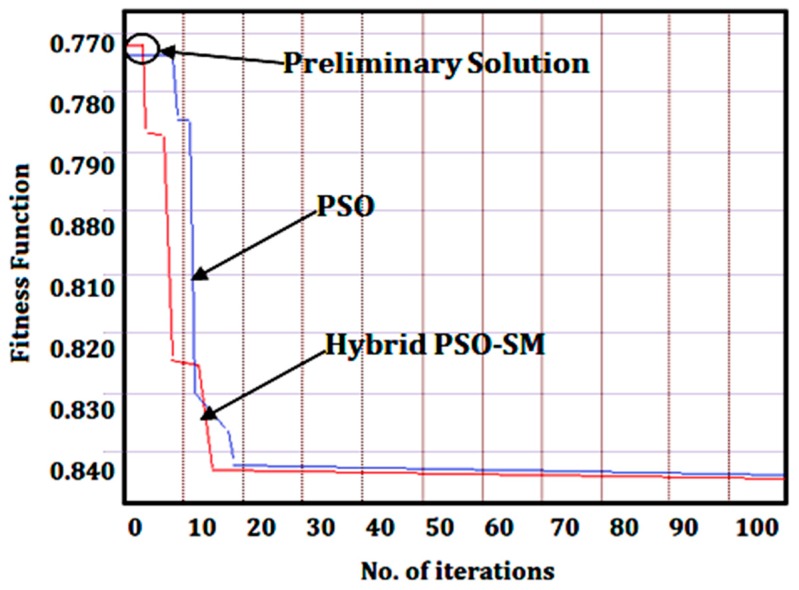
Convergence characteristics graphs for bi-objective optimization of combined objective.

In this section, the multi-objective optimization (in which more than a single factor is involved) with respect to the subjected process parameters was performed. The bi-objective optimization was performed in three manners: (1) maximization of responses, (2) minimization of responses and (3) grouping of minimization and maximization. In this work, the objective was to minimize the surface roughness and power consumption values. Hence, the minimization function was used as a fitness function in this work. The fitness function is initially developed by converting the all responses into single function and then the optimization is performed on this single objective function. The conversions of responses are made by using Equation (16):

(16) $$X_{min}=\frac{W_{1}\times X_{1}}{X_{1min}}+\frac{W_{2}\times X_{2}}{X_{2min}}$$

where, $X_{1min}$ *=* minimum value of surface roughness, $X_{2min}$ is minimum value of power consumption*, W*_1_ and *W*_2_ are the weights assigned to the responses, i.e., 0.50 for each response. This combined function $X_{\min}$ was used as an objective function in MATLAB program and the optimization was performed by considering the same boundary conditions and initial learning parameters, respectively. From the generated results in Table 9, the optimum values selected were:$\;V_{40.77\;},\;F_{0.11}$, $DOC_{0.2}$ and *f*_20_ for simultaneously minimizing (i.e., 0.8452) both the responses i.e., surface roughness and power consumption values. The convergence characteristics graph is shown in Figure 8.

#### 4.3.4. Algorithms Confirmation

To ensure the efficiency of PSO and the hybrid PSO-SM method, comparative analysis in terms of percentage error, standard deviation, success rate and running time etc. was performed. The success rate, ideal values and running time were directly achieved from the MATLAB code, whereas the percentage error and standard deviation were calculated by Equations (17) and (18):

(17) $${\%}_{error}=\left|\frac{{\#}_{Experimental}-{\#}_{Thoeretical}}{{\#}_{Thoeretical}}\right|\times 100$$

(18) $$s=\sqrt{\frac{\sum_{i=1}^{N}{\left(x_{i}-\bar{x}\right)}^{2}}{N-1}}$$

where, {*x*_1_, *x*_2_, …, *x_n_*} are the observed values, $\bar{x}$ is the mean value of these observations, *N* is the number of observations.

After that, the 100 iterations were run at optimal conditions and the average data were calculated. From the given optimized results and comparative analysis (Table 9), it was noticed that the hybrid PSO-SM method performed better than the standard PSO method in mono as well as bi-objective optimization of the UAT process parameters. The success rate was 90% and running time wass only 6 s in the case of hybrid PSO-SM algorithm. Besides, the low values of percentage error and standard deviation of hybrid PSO-SM proved the high reliability and stability of algorithm towards the global optimal solution. Similarly, the results of the standard PSO showed that the success rate was 80%, running time was 15 s, percentage error and standard deviation were high for achieving the optimal solution. The performance of the hybrid PSO-SM method was high because the initial learning parameters of PSO were improved with the simplex method which was not in the case of the standard PSO. Another relevant aspect is that the independent swarm of PSO method i.e., vector $X_{i}\;\left(N_{i},x_{i},C_{1i},C_{2i}\right)\;i\;=\;1,\ldots ,\;n\;+\;1,$ where *n* is the number of PSO parameters and *N_i_* is an integer number computed with the steps of simplex method i.e., reflection, contraction, expansion and shrinkage and with these integration steps the searching capability of swarms are increased, and as a result swarms rapidly move towards the global optimal solution. Lastly, it is worth noting that the high stability, reliability and confidence of the hybrid PSO-SM method confirmed its effectiveness during optimization of the UAT process.

## 5. Conclusions

In this work, a robust technique in determining the optimal control parameters in UAT of Nimonic-90 alloy was presented with the goal of obtaining the lowest surface roughness and power consumption values. The optimization was performed in two ways: (1) mono-objective and (2) bi-objective by using a standard PSO and a hybrid PSO-SM, respectively. Further, in-depth analysis of the process mechanism by using contour plots was performed in the Results and Discussion section. From this work, the following conclusions may be drawn:

1. The performance of the hybrid PSO-SM was better in terms of lowering the running time, error and standard deviation as compared with the standard PSO method. The fact is that the initial learning parameters of PSO were improved with the simplex method and they may have increased the performance as compared with the standard PSO.
2. The results of the mono-objective optimization method showed that the cutting speed of 61.14 m/min, feed rate of 0.11 mm/rev, depth of cut of 0.1 mm and frequency of 20 kHz were ideal parameters for surface roughness values. Similarly, the cutting speed of 27.14 m/min, a higher value of feed rate of 0.33 mm/rev, lower value of depth of cut of 0.1 mm and frequency of 20 kHz were the optimum parameters for lowering the power consumption.
3. Likewise, the results of bi-objective optimization show that the medium value of cutting speed of 40.77 m/min, a lower feed rate of 0.11 mm/rev, a medium depth of cut of 0.2 mm and frequency of 20 kHz were the best settings for simultaneously lowering the responses.
4. From the statistical analysis, it has been noticed that the feed rate was the major factor affecting the surface roughness values, whereas the cutting speed claimed the most significant terms for power consumption.
5. The contour effect plots showed that the ultrasonic assisted turning process reduced the surface roughness and power consumption values as compared with the conventional turning process. This was due to the basic reason that the ultrasonic vibration produced the micro-chipping effect and thereby resulted in low surface roughness as well as power consumption values. Besides, the chips formed during the UAT processes were regular and fragmented when compared to those obtained from the CT process.
6. With the ultrasonic assisted machining, the surface roughness was improved by 5%–10% and the power consumption was reduced from 8%–10% when we compared the results with ordinary turning.

## Figures and Tables

**Table 1:** Chemical composition of Nimonic-90.

Elements	C	Si	Mg	Cr	Ni	Ti	Al	Co	Fe
% Weight	0.08	0.13	0.018	18.1	58	2.4	1.09	18.5	0.82

**Table 2:** Cutting insert specifications.

Insert Part Number	CNMG 120408CQ
Rake angle	5°
Relief angle	0°
Nose radius	0.8 mm
Lead angle	45°
Point angle	80°

**Table 3:** Range and levels of process parameters.

Parameters	Range		
	Level 1	Level 2	Level 3
Cutting speed (m/min)	27.14	40.77	61.14
Feed rate (mm/rev)	0.11	0.22	0.33
Depth of cut (mm)	0.1	0.2	0.3
Frequency (kHz)	20	18	0 (conventional)
Amplitude (µm)	10		

**Table 4:** Design and experimental results of the L_9_ orthogonal array.

Sr. No.	Control Variables				Average Responses	
	V (m/min)	F (mm/rev)	DOC (mm)	f (kHz)	$R_{a}\;(\mu m)$	P (W)
1	27.14	0.11	0.1	20	0.37	288.67
2	27.14	0.22	0.2	18	1.56	337.33
3	27.14	0.33	0.3	0	2.21	308.33
4	40.77	0.11	0.2	0	1.06	302.67
5	40.77	0.22	0.3	20	0.9	335.67
6	40.77	0.33	0.1	18	1.14	312.67
7	61.14	0.11	0.3	18	0.64	413.67
8	61.14	0.22	0.1	0	0.67	349.67
9	61.14	0.33	0.2	20	1.26	335.00

**Table 5:** Analysis of variance of means for surface roughness.

Source	DF	Adj SS	Adj MS	F-Value	*p*-Value	%C
Cutting speed	2	1.26423	0.63211	134.6	0.001	17.051
Feed	2	3.26703	1.63351	347.83	0.002	44.065
Depth of cut	2	1.79147	0.89574	190.73	0.002	24.163
Frequency	2	1.00732	0.50366	107.25	0.000	13.586
Error	18	0.08453	0.0047			
Total	26	7.41459				

**Table 6:** Analysis of variance of means for and power consumption.

Source	DF	Adj SS	Adj MS	F-Value	*p*-Value	%C
Cutting speed	2	15,407	7703.4	20.86	0.000	39.798
Feed	2	2733	1366.3	3.7	0.045	7.0596
Depth of cut	2	6445	3222.3	8.73	0.002	16.648
Frequency	2	7483	3741.6	10.13	0.001	19.32
Error	18	6646	369.2			
Total	26	38,713				

**Table 7:** Initial parameters of PSO.

Input Parameters	Value of Parameters
S, number of agent particles	50
Number of iterations	100
Maximum permissible inertia weight	1.4
Minimum permissible inertia weight	0.5
Maximum defined learning rate, *C*_1max_ = *C*_2max_	2
Minimum defined learning rate, *C*_1min_ = *C*_2min_	1.5
H	5

**Table 8:** Initial parameters of HPSO-SM.

Input Parameters	Value of Parameters
S, number of agent particles	50
Number of iterations	100
Maximum permissible inertia weight	1.156
Minimum permissible inertia weight	1.143
Maximum defined learning rate, *C*_1max_ = *C*_2max_	1.345
Minimum defined learning rate, *C*_1min_ = *C*_2min_	1.845
H	5

**Table 9:** Control variables and their selected values (for optimal response variables).

Control Variables	Optimal Values for Response Variables					
	Surface Roughness (µm)		Power Consumption (Watts)		Combined Values	
	PSO	HPSO-SM	PSO	HPSO-SM	PSO	HPSO-SM
Cutting speed (m/min)	61.14	61.14	27.14	27.14	40.77	40.77
Feed (mm/rev)	0.11	0.11	0.33	0.33	0.11	0.11
Depth of cut (mm)	0.1	0.1	0.1	0.1	0.2	0.2
Frequency (kHz)	20	20	20	20	20	20
Best solution	<0.35	>0.35	<270	>270	<0.8452	>0.8452
Mean solution	0.353	0.350	272.33	270.52	0.8572	0.8456
Standard deviation	0.458	0.352	0.583	0.383	0.522	0.324
Average time (s)	15	6	15	6	15	6
Success rate	80	90	80	90	80	80
Percentage error	5.34	1.24	6.3	1.5	6.34	1.4

## References

[1] Gupta M.K., Sood P.K. (2017). Machining comparison of aerospace materials considering minimum quantity cutting fluid: A clean and green approach. Proc. Inst. Mech. Eng. Part C J. Mech. Eng. Sci..

[2] Rao M.S., Venkaiah N. (2015). Parametric Optimization in Machining of Nimonic-263 Alloy using RSM and Particle Swarm Optimization. Procedia Mater. Sci..

[3] Ghoreishi R., Roohi A.H., Ghadikolaei A.D. (2019). Evaluation of tool wear in high-speed face milling of Al/SiC metal matrix composites. J. Braz. Soc. Mech. Sci. Eng..

[4] Jang D.Y., Jung J., Seok J. (2016). Modeling and parameter optimization for cutting energy reduction in MQL milling process. Int. J. Precis. Eng. Manuf. - Green Technol..

[5] Sharma V., Pandey P.M. (2016). Optimization of machining and vibration parameters for residual stresses minimization in ultrasonic assisted turning of 4340 hardened steel. Ultrasonics.

[6] Sajjady S.A., Abadi H.N.H., Amini S., Nosouhi R. (2016). Analytical and experimental study of topography of surface texture in ultrasonic vibration assisted turning. Mater. Des..

[7] Sofuoğlu M.A., Çakır F.H., Gürgen S., Orak S., Kuşhan M.C. (2018). Numerical investigation of hot ultrasonic assisted turning of aviation alloys. J. Braz. Soc. Mech. Sci. Eng..

[8] Puga H., Grilo J., Oliveira F.J., Silva R.F., Girão A.V. (2019). Influence of external loading on the resonant frequency shift of ultrasonic assisted turning: Numerical and experimental analysis. Int. J. Adv. Manuf. Technol..

[9] Zhang C., Ehmann K., Li Y. (2015). Analysis of cutting forces in the ultrasonic elliptical vibration-assisted micro-groove turning process. Int. J. Adv. Manuf. Technol..

[10] Ahmed N., Mitrofanov A.V., Babitsky V.I., Silberschmidt V.V. (2007). Analysis of forces in ultrasonically assisted turning. J. Sound Vib..

[11] Maurotto A., Roy A., Babitsky V.I., Silberschmidt V.V. (2012). Analysis of Machinability of Ti- and Ni-Based Alloys. Advanced Materials and Structures IV.

[12] Maurotto A., Muhammad R., Roy A., Silberschmidt V.V. (2013). Enhanced ultrasonically assisted turning of a β-titanium alloy. Ultrasonics.

[13] Silberschmidt V.V., Mahdy S.M., Gouda M.A., Naseer A., Maurotto A., Roy A. (2014). Surface-roughness Improvement in Ultrasonically Assisted Turning. Procedia Cirp.

[14] Zhong Z.W., Lin G. (2006). Ultrasonic assisted turning of an aluminium-based metal matrix composite reinforced with SiC particles. Int. J. Adv. Manuf. Technol..

[15] Nath C., Rahman M. (2008). Effect of machining parameters in ultrasonic vibration cutting. Int. J. Mach. Tools Manuf..

[16] Vivekananda K., Arka G.N., Sahoo S.K. (2014). Finite Element Analysis and Process Parameters Optimization of Ultrasonic Vibration Assisted Turning (UVT). Procedia Mater. Sci..

[17] Gupta M.K., Sood P.K., Sharma V.S. (2016). Optimization of machining parameters and cutting fluids during nano-fluid based minimum quantity lubrication turning of titanium alloy by using evolutionary techniques. J. Clean. Prod..

[18] Raju M., Gupta M.K., Bhanot N., Sharma V.S. (2019). A hybrid PSO–BFO evolutionary algorithm for optimization of fused deposition modelling process parameters. J. Intell. Manuf..

[19] Singh G., Gupta M.K., Mia M., Sharma V.S. (2018). Modeling and optimization of tool wear in MQL-assisted milling of Inconel 718 superalloy using evolutionary techniques. Int. J. Adv. Manuf. Technol..

[20] Khanna N., Davim J.P. (2015). Design-of-experiments application in machining titanium alloys for aerospace structural components. Measurement.

[21] Khafaji S.O.W., Manring N. (2019). Sensitivity analysis and Taguchi optimization procedure for a single-shoe drum brake. Proc. Inst. Mech. Eng. Part C J. Mech. Eng. Sci..

[22] Chen F.C., Huang H.H. (2006). Taguchi-fuzzy-based approach for the sensitivity analysis of a four-bar function generator. Proc. Inst. Mech. Eng. Part C J. Mech. Eng. Sci..

[23] Shokrani A., Dhokia V., Newman S.T. (2016). Investigation of the effects of cryogenic machining on surface integrity in CNC end milling of Ti–6Al–4V titanium alloy. J. Manuf. Process..

[24] Islam M.N., Pramanik A. (2016). Comparison of Design of Experiments via Traditional and Taguchi Method. J. Adv. Manuf. Syst..

[25] Ezilarasan C., Kumar V.S., Velayudham A., Palanikumar K. (2011). Modeling and analysis of surface roughness on machining of Nimonic C-263 alloy by PVD coated carbide insert. Trans. Nonferrous Met. Soc. China.

[26] Makadia A.J., Nanavati J.I. (2013). Optimisation of machining parameters for turning operations based on response surface methodology. Measurement.

[27] Bhushan R.K. (2013). Optimization of cutting parameters for minimizing power consumption and maximizing tool life during machining of Al alloy SiC particle composites. J. Clean. Prod..

[28] Gupta M.K., Sood P.K., Sharma V.S. (2015). Machining Parameters Optimization of Titanium Alloy Using Response Surface Methodology and Particle Swarm Optimization Under Minimum Quantity Lubrication Environment. Mater. Manuf. Process..

[29] Sahu N.K., Andhare A.B. (2018). Multiobjective optimization for improving machinability of Ti-6Al-4V using RSM and advanced algorithms. J. Comput. Des. Eng..

[30] Sathish T. (2018). BCCS Approach for the Parametric Optimization in Machining of Nimonic-263 alloy using RSM. Mater. Today Proc..

[31] Youssef H.A., El-Hofy H. (2008). Machining Technology: Machine Tools and Operations.

[32] Chen L., Monteiro T., Wang T., Marcon E. (2019). Design of shared unit-dose drug distribution network using multi-level particle swarm optimization. Health Care Manag. Sci..

[33] Ameur T., Assas M. (2012). Modified PSO algorithm for multi-objective optimization of the cutting parameters. Prod. Eng..

[34] Garg A., Tai K., Lee C.H., Savalani M.M. (2014). A hybrid M5 -genetic programming approach for ensuring greater trustworthiness of prediction ability in modelling of FDM process. J. Intell. Manuf..

[35] Gaitonde V.N., Karnik S.R. (2012). Minimizing burr size in drilling using artificial neural network (ANN)-particle swarm optimization (PSO) approach. J. Intell. Manuf..

[36] Nie R., Yue J.H., Deng S.Q. Hybrid particle swarm optimization-simplex algorithm for inverse problem. Proceedings of the 2010 Chinese Control and Decision Conference.

[37] Begambre O., Laier J.E. (2009). A hybrid Particle Swarm Optimization—Simplex algorithm (PSOS) for structural damage identification. Adv. Eng. Softw..

[38] Fan S.-K.S., Zahara E. (2007). A hybrid simplex search and particle swarm optimization for unconstrained optimization. Eur. J. Oper. Res..

[39] Zahara E., Kao Y.-T. (2009). Hybrid Nelder–Mead simplex search and particle swarm optimization for constrained engineering design problems. Expert Syst. Appl..

[40] Xu Y., Zou P., He Y., Chen S., Tian Y., Gao X. (2016). Comparative experimental research in turning of 304 austenitic stainless steel with and without ultrasonic vibration. Proc. Inst. Mech. Eng. Part C J. Mech. Eng. Sci..

[41] Parsopoulos K.E., Vrahatis M.N. (2002). Recent approaches to global optimization problems through Particle Swarm Optimization. Nat. Comput..

